# The Quiet Surgeon: A Qualitative Analysis of the Introverted Experience Throughout a Career in Academic Surgery

**DOI:** 10.1097/AS9.0000000000000685

**Published:** 2026-05-29

**Authors:** Rebecca Tang, Sarah E. Rudasill, Peter Stehr, Jonathan B. Greer, Cornelia L. Griggs, Genevieve M. Boland, Roy Phitayakorn, Dandan Chen, Sophia K. McKinley

**Affiliations:** From the * Department of Surgery, Massachusetts\General Hospital, Boston, MA; † Harvard Medical School, Boston, MA.

**Keywords:** academic surgery, introversion, leadership, professional development, professional identity formation

## Abstract

**Objective:**

This study seeks to describe the lived experiences of self-identified introverts throughout a career in academic surgery.

**Summary Background Data:**

Surgeons and leaders are often perceived as extroverted, with the belief that introverts must project extroversion to succeed as surgeons or leaders. However, many surgical trainees entering academic surgery identify as introverted.

**Methods:**

Semi-structured interviews were conducted with self-identified introverted general surgery residents and attending surgeons at a tertiary academic medical center in the Northeast. Participants were purposively sampled to include junior and senior residents, early- and late-career attendings, and men and women participants. Interview transcripts were iteratively analyzed using inductive thematic analysis with a constructivist approach to develop a theoretical framework describing the experience of introverts in academic surgery.

**Results:**

Nine surgical residents and 8 attending surgeons participated in interviews. Four overarching themes were identified: (1) the lived experiences and self-perceptions of introverts are highly individual; (2) introverts must reconcile their preferences with the necessity of extroverted behaviors in academic surgery; (3) introverted preferences for relationship-building often conflict with the interpersonal demands of a career in academic surgery; and (4) introverts evolve over a surgical career through both internally and externally motivated strategies.

**Conclusions:**

Introverts experience challenges surrounding the sociocultural conventions inherent to professional advancement in academic surgery. Over time, introspection, paired with support from role models and mentors, permits introverts to demonstrate their value and strengths. This study highlights opportunities for supporting the growth and development of introverts in hopes of fostering greater inclusivity within academic surgery.

## INTRODUCTION

The culture of surgery has long been associated with confidence, decisiveness, and leadership—traits commonly ascribed to extroverted personalities. Introversion and extroversion, first described by psychologist Carl Jung, reflect differences in how individuals direct their energy.^1^ Extroverts tend to draw energy from social interaction, process information through group discussion, make decisions quickly, and more comfortably take risks. In contrast, introverts often recharge through solitude, prefer independent work, and approach decision-making with deliberation.^2,3^ In the United States, extroversion is often equated with leadership potential and professional success,^2,4^ and extroverts are disproportionately represented among workforce leaders.^5–9^ However, the idealization of extroversion risks overlooking the strengths of introversion and the value of diverse personality types within high-performance environments.^10^

Within academic surgery, the extrovert ideal is especially salient.^11–13^ Surgical culture has traditionally emphasized extroverted behaviors, and the American College of Surgeons identifies leadership as a core surgical professional competency.^14^ However, the landscape of surgery may be shifting: nearly 70% of surgical trainees identify as introverted in 1 study.^15^ As introverts rise through the ranks of academic surgery, they may encounter challenges such as people exhaustion, a relentless work pace, frequent interruptions, pressure to self-promote, demands for team-based work, and negative impressions.^16^ These challenges may be amplified in surgical environments that reward outward confidence, social engagement, and extroverted leadership styles.^17,18^ Currently, the experiences of introverted academic surgeons remain poorly characterized. This exploratory qualitative study seeks to describe the lived experience of self-identified introverts throughout a career in academic surgery, delineate the advantages and challenges of introversion across domains of surgical practice, and identify strategies for mitigating any identified challenges.

## METHODS

All study materials and procedures were reviewed and approved by the Mass General Brigham Institutional Review Board (Protocol #2024P003543). The COnsolidated criteria for REporting Qualitative research (COREQ) were followed for study design and reporting.^19^

### Participants and Recruitment

Any general surgery resident or attending surgeon at 1 tertiary academic medical center who self-identified as introverted was eligible to participate in the study. Participants were recruited via snowball sample. Initial participants were identified based on prior self-initiated disclosures of self-identified introversion. At the end of their interviews, participants were asked to recommend any individuals who might meet inclusion criteria; these individuals were then invited by email to participate and were purposively sampled for diversity in gender and career stage (Supplemental Figure 1 https://links.lww.com/AOSO/A624). Study participants were provided a $50 gift card as partial remuneration of their time.

### Data Collection

A semi-structured interview guide was developed based on literature review^10,16^ and reviewed by 3 members of the research team: a surgical resident (R.T.), an education researcher with experience in educational psychology (D.C.), and a surgical attending with expertise in surgical education (S.K.M.). Representative questions from the semi-structured interview guide are provided in Supplemental Figure 2 https://links.lww.com/AOSO/A625. Participants were asked to reflect on their relationship with being an introvert, advantages or disadvantages of introversion in surgery, perceived impacts on their past or future career trajectories, and advice for other introverts and their supervisors. Participants were also asked to provide their demographics and years of experience. Post-graduate year (PGY) was defined as years of clinical training. The interview guides were piloted on 2 residents and 2 attending surgeons and reviewed by the study team. As only minor adjustments were made to the interview guides, data from the pilot interviews were included in our analysis.

Interviews were conducted from December 2024 through April 2025 by a research team member with previous experience and training in qualitative research methods and no supervisory or evaluative role over participants (R.T.). Prior to each interview, participants were informed of the study purpose and provided verbal consent. Interviews were conducted virtually, video-recorded, and transcribed using Zoom (Version 6.3.11). At the end of each interview, the interviewer summarized key ideas to allow participants an opportunity to modify the researcher’s understanding of the interview content. Field notes were recorded by the interviewer after each interview. Transcripts were reviewed, deidentified by the interviewer, and returned to participants for review. Interviews were continued until 3 consecutive interviews generated no new analytic codes.

### Data Analysis

Interview transcripts were iteratively analyzed using inductive thematic analysis with a constructivist approach.^20^ This methodology was selected as review of the literature did not reveal any existing theoretical frameworks related to the experience of introverts in surgery. Additionally, constructivism is well suited to investigate the diverse and heterogenous perspectives of individuals without the assumption of a single, universal truth.^21^ All transcripts were independently coded by 2 members of the research team (R.T., S.E.R.) using Dedoose (Version 10.0.025). Regular meetings for iterative consensus-building and refinement of the codebook were held between 4 members of the research team (R.T., S.E.R., P.S., S.K.M.). These codes were organized into themes, and a constructivist grounded theory approach was used to develop a theoretical framework.^22^ The themes and theoretical framework were iteratively refined by a multidisciplinary team (R.T., S.E.R., P.S., S.K.M., G.B., R.P., D.C.) before being shared with a convenience sample of 2 surgical residents and 2 attending surgeon participants for further review and member checking. No revisions were requested by the participants.

The research team engaged in formal reflexivity to minimize bias by examining how each member’s personal beliefs and experiences might influence the research process. Notably, the team deliberately included both introverts and extroverts, as well as diversity of gender and professional experience: R.T. (introverted woman surgical resident), S.E.R. (introverted woman surgical resident), P.S. (extroverted man medical student), S.K.M. (introverted woman surgical attending), R.P. (introverted man surgical attending), G.B. (extroverted woman surgical attending), and D.C. (introverted woman education researcher). Representative quotations in this manuscript have been edited for readability and labeled “R” for residents or “A” for attendings, followed by interview order.

## RESULTS

Study participant demographics are displayed in Table 1. Nine surgery residents and 8 attending surgeons participated in interviews. Among surgery residents, 5 (55.6%) were women, 4 (44.4%) were non-White, 4 (44.4%) were junior residents (defined as PGY 1 or 2), and 5 (55.6%) were senior residents (defined as PGY 3 or higher). Among attending surgeons, 4 (50.0%) were women, 3 (37.5%) were non-White, 3 (37.5%) were junior attendings (defined as 15 or fewer years in practice), and 5 (62.5%) were senior attendings (>15 years in practice). Surgical attendings interviewed included academic surgical leaders such as past or present presidents of national societies, division chairs, and education program leaders. Median interview duration was 37 minutes (range 24–56 minutes).

**TABLE 1.** Participant Demographics

Resident (n = 9)	Attending (n = 8)	Attending (n = 8)
Gender		
Man	4 (44.4%)	4 (50.0%)
Woman	5 (55.6%)	4 (50.0%)
Race		
White	5 (55.6%)	5 (62.5%)
Non-White	4 (44.4%)	3 (37.5%)
Experience		
PGY 1–2	4 (44.4%)	
PGY 3–5	5 (55.6%)	
YIP ≤15		3 (37.5%)
YIP >15		5 (62.5%)

PGY indicates post-graduate year, YIP, years in practice.

All 16 codes appeared in at least 2 interviews, indicative of thematic saturation. These codes were collapsed into 4 overarching themes: (1) the lived experiences and self-perceptions of introverts are highly individual, (2) introverts must reconcile their preferences with the necessity of extroverted behaviors in academic surgery, (3) introverted preferences for relationship-building often conflict with the interpersonal demands of a career in academic surgery, and (4) introverts evolve over a surgical career through both internally and externally motivated strategies. Tables 2–5 display codebooks with themes, codes, and exemplar quotes.

**TABLE 2.** Codes and Exemplar Quotes for Theme 1

Code	Exemplar Quotes
Theme 1. Exploring the lived experiences and self-perceptions of introverts	
Defining introversion	“I think that the definition of introversion is people who get their energy from being alone and not necessarily from other people.” – A5 “I’ve never actually really considered myself shy or timid, which I think is conflated with introversion.” – R3
Behavior versus personality	“There’s a difference between being extroverted, like being loud and being talkative, and truly being an extrovert.” – A2 “I have very extroverted tendencies at work. If you’re the [leader], you need to be pretty vociferous and active and out there… But it doesn’t mean I’m not an introvert still, because it’s what I do when I’m done with my task, when I go home at night.” – A6 “I think it’s really hard to pin down a surgeon’s personality on the outside. I think you have to be confident in who you are to do this job, to take care of people. And so with that in mind, I think with surgeon leaders, you never really know what they are on the inside.” – R8
Feelings about being introverted	“Personally, I’m very happy with being introverted and I look forward to my alone time.” – R5 “For most of my life, it’s something that I’ve had to overcome.” – R9 “It’s just something different and it works for you or it doesn’t. I don’t know that it’s an advantage or a disadvantage.” – A5
Introversion is an individual experience	“I think this is a very multifactorial and intersectional topic… People have different ‘version’ orientations… A one size fits all approach does not work.” – R2 “I’m not sure where the intersection is of culture and introversion, extroversion… it is an interesting intersection, because there’s cultural stereotypes, but there’s also cultural norms that feed into those stereotypes.” – A8
Introspection	“I feel like my introvertedness is what has made me as successful as I am, because I have a lot of thoughtful reflection, so the things that I do are very purposeful and methodical.” – A4 “Being introverted, I think you’re naturally more focused inward, and I think it takes a lot of self-reflection to get better and do better every day as a surgeon.” – R8

**TABLE 3.** Codes and Exemplar Quotes for Theme 2

Code	Exemplar Quotes
Theme 2. Reconciling introverted preferences and the necessity of extroverted behaviors in academic surgery	
Processing information	“I’m the type of person who wants to have information, let it sit for a bit, process it, and then come up with an answer.” – R6 “Sometimes something will come up and they’ll ask me and give me a time to speak, and my mind will be totally blank. And then ten minutes later, I’ll be like, oh no, I have all these things I want to say. So yes, knowing ahead of time is helpful.” – A5
Getting work done	“One of the advantages to being an introvert is I can, with total impunity, lock myself away somewhere alone and get all of the work that I need to get done, done and not feel internal pressure to go be extroverted.” – R9 “I used to think it was an advantage not to go out, because then I would get work done.” – A5 “I like to work around other people, but I like for it to be generally on the quieter side… I find it harder to do more thinking tasks when there’s a lot of other activity going on.” – R4
Extrovert ideal in academic surgery	“You know what’s really weird? Nobody ever asks you to be an introvert. It’s always like you’re asked to be the extrovert, so to speak.” – A2 “In surgery residency, even if you take a stab at something and you’re wrong, if you say it confidently… people who do that tend to be seen as more competent.” – R7 “Getting ahead in academic surgery is easier or works better if you are more social… There is no doubt that at least at the national level, people do have to accept that academics are based probably a little bit too much on who you know, how good of a socializer you are.” – A7
Impact on career choices	“I initially came into medical school not thinking surgery because I just assumed that only extroverted people went into it… The more variety of people I saw who were surgical attendings that I looked up to, the more I realized that this was something that I could do.” – R5 “I do feel like it probably limits my desire to very quickly go into leadership roles. But if I decide to go into one, then it would be for a very specific purpose rather than just being for a flashy title.” – A4

**TABLE 4.** Codes and Exemplar Quotes for Theme 3

Code	Exemplar Quotes
Theme 3. Contrasting the enjoyment of deeper relationship-building against the difficulties of navigating group dynamics	
Social battery	“Some days, the actual job is not difficult. I come home exhausted, drained from all the interactions… Medicine is so social. You’re always interacting and it’s just an additional thing that drains energy when you’re already tired.” – R6 “When I don’t have ample time off to recharge, I find it really difficult. I honestly haven’t found anything that can quickly or easily recharge that battery.” – R9 “I’ve started just blocking off times in my day where I won’t let people schedule meetings.” – A5
Relationship-building	“It always takes me at least a year to kind of get comfortable and feel like I know people well enough.” – A1 “Sometimes it can be hard to click with attendings when you’re not super talkative. So that’s been something difficult in residency.” – R1 “If they’re more comfortable with you, maybe they’ll let you do more in the OR, or maybe they’ll seek you out more for research opportunities.” – R7 “Although I don’t make as many connections, I feel like the connections that I do make are a lot stronger and a lot deeper.” – R9 “A real strength of being an introvert is that I have quite good situational awareness and emotional awareness.” – R1
Participation in group settings	“I do feel like I get spoken over quite a bit.” – A1 “The larger the group and the more people that are senior to me, then the less I’m able to speak up.” – R9 “I used to feel like I was screaming when I was a junior because so much of my feedback was, You should speak with more confidence and speak up.” – R3 “I feel more conscious of the comment that I’m going to make, whether it makes sense or if someone already said it. If I’m in a group setting and I’m going to comment something, I feel like it has to be more thoughtful.” – R6 “Maybe it’s because I don’t say a lot. So when I do speak, people are like, oh, I wanna hear what [name] has to say.” – A6
Dynamics of relationships with extroverts	“I’ve learned the minimum expectation that others need, to feel like you’re not an ice queen.” – R3 “If your boss is a hard-driving extrovert, and you know you need to have a difficult conversation with him or her, that’s another potential barrier. Maybe it’s not a barrier for an extrovert, but it’s a barrier for an introvert subordinate compared to an extrovert.” – A8 “For people who are more introverted… it’s really helpful to pair up with an extrovert.” – A4

**TABLE 5.** Codes and Exemplar Quotes for Theme 4

Code	Exemplar Quotes
Theme 4. Fostering personal growth and success over a surgical career through both individual and environmental adaptations	
Improving the learning environment for introverts	“I think that onus is not on the resident, but on the people that they work with to recognize that there’s many different personality types of people that go into this field.” – A2 “If they could be a little bit more open-minded about differences in personalities and behaviors, and that some people are on the quieter side, but that doesn’t mean they’re any worse than other residents.” – R6 “Give people the time and space that they need to really be able to respond properly.” – A4
Expanding the comfort zone	“If you realize this about yourself, it’s an asset. Stay true to yourself and don’t change. Figure out how you want to do this career long-term, but recognize that at the beginning, you’re going to have to put yourself out there in ways that don’t feel comfortable... The reality of the system is that if you don’t try to stretch yourself a little, you might miss out on opportunities. You have to push yourself a little out of your comfort zone.” – A1 “I know that if I’m going to be put on a committee or be part of an organization… I have to push myself.” – R3 “I know it’s a harsh view of things, but nobody’s going to advocate for you, right? You have to advocate for yourself.” – A4
Adaptation and growth over time and medical training	“I’ve had to become more extroverted at times related to leading teams, to speaking up, to making decisions.” – R2 “I think you naturally progress and you become naturally comfortable interacting with people more.” – R8 “It’s a work in progress.” – A2

Theme 1: The lived experiences and self-perceptions of introverts are highly individual

Participants defined introversion as deriving energy from time spent alone rather than with groups of people, sometimes conflated with—but distinct from—shyness and social anxiety. Participants identified introspection, thoughtfulness, self-knowledge, and self-sufficiency as key strengths of introversion. Importantly, participants distinguished behaviors influenced by personality and preferences from objective performance and ability. For instance, an introvert’s preferences for avoiding public speaking and excessive socializing did not equate to an inability to perform well at these extroverted tasks. As a result, participants noted the difficulty of identifying whether their colleagues were introverted or extroverted. As 1 participant reported, “I think a lot of people I work with see me as more extroverted than I see myself, which has been a process of trying to project ‘extrovertedness’ in my work” (A4).

Participants identified diverse behaviors, preferences, and attitudes reflecting the highly individual experience of introversion. Personal experiences were also shaped by intersections with factors such as gender, cultural upbringing, and socioeconomic background. Although most participants considered introversion a neutral or positive trait, several noted that society undervalues introverted traits: “I think of it as a positive, but society thinks of it as negative… I think it would be easier if I were not introverted” (R7).

Theme 2: Introverts must reconcile their preferences with the necessity of extroverted behaviors in academic surgery

Participants reported a mismatch between their preferred ways of processing information or completing tasks and the structural and cultural conventions of surgical workflow. Most participants valued time to process information before decision-making but felt pressured to respond rapidly to new information. These included both clinical scenarios, such as managing trauma activations or decompensating patients, as well as educational activities involving Socratic teaching or “cold calling”. Many shared the sentiment that “a style of being put on the spot is not my best style of learning” (R4). Similarly, although participants considered focus and productivity as strengths of introversion, they reported limited access to quiet and semi-private workspaces conducive to these strengths.

These mismatches contributed to a broader belief that success in academic surgery required extroverted behaviors, placing introverts at a disadvantage relative to their extroverted peers. Almost all participants described experiencing pressure to socialize, network, and self-promote to an extent that felt uncomfortable and sometimes inauthentic for the purposes of academic promotion and advancement in professional societies. Participants additionally noted that conflating a confident disposition with clinical competence could lead to unfairly poor evaluations of introverted trainees, with one attending acknowledging, “I think that introverts are frequently not recognized for being as smart or as capable… When you think about ranking residents, a lot of it is based on personality” (A6).

Importantly, participants’ association of academic surgery with extroverted qualities began as early as medical school. Most participants reported perceiving surgeons as extroverted, sparking doubts about pursuing surgery as a specialty. These concerns were ultimately outweighed by passion for surgery and positive mentorship, but study participants indicated that their introverted tendencies influenced their choice of subspecialty and desire to pursue leadership roles and other opportunities for academic advancement.

Theme 3: Introverted preferences for relationship-building often conflict with the interpersonal demands of a career in academic surgery

Beyond the cultural conventions of academic surgery, participants felt challenged by the sheer volume of interpersonal interactions required in surgical practice. Most referenced a “social battery” drained by social interaction and difficult to recharge amid the demands of residency. Attendings described similar feelings of “people exhaustion” but benefited from greater agency to arrange their schedules to recharge throughout the day.

Introverts particularly struggled to be adequately heard in group settings and described a higher threshold to voice opinions. Participants found it easier to speak up when they had subject matter expertise, a meaningful contribution, or familiarity or seniority within the group. Participants also appreciated direct elicitation of their opinions, preferably with advance notice. Despite speaking less frequently, introverts felt that their words often carried more weight when they elected to speak up. Participants also frequently used asynchronous communication: “Sometimes I just have to grab people either before or after the meeting or send them emails afterwards” (A5).

In contrast to navigating group dynamics, participants identified one-on-one relationship-building as a strength of introversion. Perceptiveness, situational awareness, and emotional intelligence were reported to facilitate deeper connections and translate to increased empathy and rapport with patients. However, residents struggled with rapidly establishing connections and familiarity with attending surgeons through small talk and worried that this might result in less operative autonomy or fewer research opportunities. As 1 resident speculated, “I think it impacted my performance and evaluations… Maybe if I schmoozed better in the OR, I would have been taught better” (R1).

Interestingly, participants experienced a wide range of dynamics between introverts and extroverts. Introverts feared being misunderstood by extroverted colleagues as cold, unapproachable, unengaged in the conversation, or uninterested in relationship-building. Some participants worried that introverted subordinates may feel particularly uncomfortable voicing concerns to an extroverted superior. However, several participants felt that extroverted mentors and peers complemented introverts in a mutually beneficial relationship.

Theme 4: Introverts evolve over a surgical career through both internally and externally motivated strategies

Despite the challenges of being introverted in academic surgery, participants reported adapting over the course of their careers. Whereas junior residents primarily identified difficulties navigating extroverted environments, senior residents described personal growth through these challenges, and attending surgeons uniformly expressed career satisfaction and appreciation for the way introversion had shaped their paths. Importantly, this growth occurred both through passive experience over time and deliberate practice guided by self-reflection.

Participants identified several strategies for improving the learning environment for introverts, centered around a top–down approach to inclusivity. “Particularly with the hierarchy in surgery, you have to be the one, when you’re more senior, to bridge and reach out to more junior people” (R3). These strategies included normalizing introverted behaviors and personalities, breaking down social barriers, and creating time and space for introverts. In conjunction with improving the learning environment, participants also encouraged introverts to actively expand their comfort zone. As 1 participant remarked, “If you’re always comfortable, there’s probably something wrong with that” (A8). As traditionally extroverted behaviors like public speaking, networking, self-promotion, and self-advocacy are important for professional success, participants emphasized the importance of developing these skills like any other competency. Advice from participants for other introverts and their supervisors is summarized in Table 6.

**TABLE 6.** Recommendations for Introverts in Academic Surgery and Their Supervisors

Advice for Introverts	Advice for Supervisors of Introverts
• Identify personal strengths that are associated with introversion. • Acknowledge areas for improvement, adaptation, and growth throughout your academic career. • Find quiet spaces for working within the hospital. • Communicate your needs and preferences to avoid misunderstandings. • Use asynchronous communication such as follow-up emails or one-on-one meetings to supplement communication in group settings. • Expand your comfort zone by practicing public speaking and self-advocacy. Set discrete goals to achieve your career objectives. • Seek out introverted role models and establish a mentorship team that includes both introverts and extroverts. Consider serving as a role model or mentor for more junior introverts.	• Recognize the diversity of personality types in surgery and normalize introverted preferences. Introverts may be less likely to voice opinions, self-advocate, or self-promote. This does not equate to lack of confidence, competence, or interest. • Evaluate team members in multiple contexts. Performance in large group settings alone may not reflect an introvert’s strengths as a physician. • When possible, give introverts time to process information before expecting a reply. Give introverts advanced notice and directly solicit their opinion if you want them to speak at a meeting. • Create opportunities for asynchronous communication (eg, separate one-on-one meetings or email conversations) outside of group meetings. • When possible, advocate for presence of quiet or semi-private workspaces in the hospital for trainees.

### Progression of an Introvert Through a Career in Academic Surgery

**FIGURE 1.**
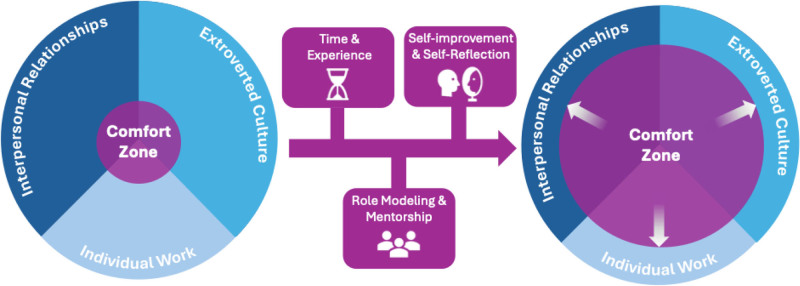
Progression of an introvert through a career in academic surgery.

Based on the results of this study, we developed a theoretical framework representing an introvert’s progression throughout a career in academic surgery (Figure 1). Through time and experience, mentorship and role modeling, and self-improvement through self-reflection, the introvert’s comfort zone grows in their academic surgical practice.

## DISCUSSION

Although introversion has been discussed in the business and leadership literature, this is possibly the first exploratory qualitative study to investigate introversion in the context of surgery. We described the lived experience of introverts at various stages of a career in academic surgery, recognizing the highly individual nature of each introvert’s experiences. We also identified commonly reported strengths and challenges of introversion in academic surgery and proposed a theoretical framework to explain the process by which introverts grow and adapt over the course of their careers. These results are consistent with the broader introversion literature, with implications for surgical training and professional development. Additionally, these results align with pre-existing educational and social theories of professional identity formation and dramaturgy.

Study participants felt pressure to project extroversion due to the perception that academic surgery rewards extroverted personalities. This reflects the “extrovert ideal” presented by Cain as “the omnipresent belief that the ideal self is gregarious, alpha, and comfortable in the spotlight.”^10^ That an extrovert ideal similarly manifests in academic surgery is unsurprising in a field that stereotypically attracts self-confident, vocal, and action-oriented trainees^23^ and prizes teamwork and leadership.^24^ Many introverted participants therefore questioned their sense of belonging, an experience shaped by intersectional sociocultural factors including gender, cultural upbringing, and socioeconomic background. Together with prior work demonstrating the influence of personality stereotypes on specialty choice^25–27^ and gender, racial, and ethnic disparities in surgical subspecialties,^28^ these findings indicate that introversion may similarly influence recruitment, particularly among trainees underrepresented in surgery. Importantly, this contributes to stereotype threat, in which the risk of confirming negative stereotypes about group membership—such as the belief that the surgical personality is extroverted—can impair performance by fostering anxiety and excessive self-regulation.^29^ In our study, such stereotype threat was mitigated by role modeling, which has been shown to affect specialty choice and improve recruitment of underrepresented groups in surgery.^30–32^ Prior research shows that surgical residents and attendings underestimate their positive influence on trainees, indicating that all introverted surgeons—despite any personal challenges—can encourage introverted medical students to pursue surgery simply by being visible and authentic.^33,34^

Beyond influencing specialty choice, perceptions of introversion also appeared to shape the educational experiences of trainees. Participants reported a detriment to the quality of education received by introverted residents, with negative impacts on both resident evaluations and operative autonomy. The role of gender, racial, and ethnic biases in resident evaluations has been well established,^35–37^ with additional studies demonstrating similar disparities in operative volume^38^ and milestone evaluations at graduation.^39^ As graduate medical education increasingly adopts competency-based models with operative and clinical autonomy dependent upon faculty evaluations, introverted residents may progress more slowly due to limited self-advocacy and educators’ conflation of competence with confidence. Faculty education on these potential biases may provide more equitable training experiences.

Introverted participants described incorporating more extroverted behaviors despite maintaining introverted preferences. This reflected both increasing comfort over time and intentional behavioral adaptations. Interestingly, the self-reflection, self-knowledge, and self-regulation inherent to introversion may facilitate these adaptations. Although this process of self-reflection appeared to be self-initiated by participants, there may be opportunities for externally initiated reflection through educational programming. Interventions such as protected process groups with structured reflection and discussion have been shown to be feasible and positively reviewed.^40^ As reflection is 1 of the 4 key pillars of Kolb’s experiential learning cycle,^41^ introverted and extroverted residents alike would benefit from opportunities to reflect on their professional development.^42^ Finally, learning extroverted behaviors, as with any skill, requires introverts to leave their comfort zone. Perhaps one of the greatest pitfalls for introverts navigating academic surgery is the temptation to resist uncomfortable growth processes. Educators and mentors may facilitate these processes by creating psychologically safe environments, allowing introverts to speak up and take risks without fear of judgment.^43^ Targeted coaching may further help introverts achieve their professional goals.

The tension between personal preferences and professional demands presented a source of stress and can be understood as identity dissonance within the theoretical framework of professional identity formation. Professional identity is defined as “the integration of personal values, morals, and attributes with the norms of the profession.”^44^ The surgical professional identity traditionally includes confidence, assertiveness, and team leadership—traits associated with extroversion.^23,24^ Surgical professional identity formation represents a dynamic socialization process shaped by individual traits and life experiences, interpersonal relationships within multidisciplinary teams, beliefs about the surgeon’s role, external influences such as practice setting and culture, and professional experiences and reflection.^45–47^ Divergence between personal identities and perceived professional ideals results in identity dissonance, which can hinder success and increase burnout.^48,49^ Participants described adapting their outward behaviors to meet these expectations while maintaining introverted preferences. This process aligns with the broader concepts of professional socialization and norm conformity, in which individuals modify behaviors to fit the cultural expectations of a field.^50^ In our theoretical framework, behaviors outside an individual’s comfort zone may initially reflect such performance but can, over time, become integrated into one’s professional identity (Fig. 1). Advice from study participants (Table 6) may support introverts in professional identity formation.

There are several limitations to our study. First, the results of this study must be interpreted within the context of single, highly selective tertiary academic institution with a large residency program in a prominent Northeastern city. This environment represents an exceptionally competitive and distinctive training setting, with rigorous selection processes for both residents and faculty. As such, although the study setting strongly embodies academic surgery, our findings may not reflect the experiences of introverted surgeons in private practice or community settings. Additionally, the single-institution design limits our understanding of the impact of regional or institutional culture and the transferability of our findings. Second, our study population predisposes our findings to survivorship bias, as participants had already selected surgery and matched to train at a highly ranked academic institution. Our study therefore does not capture the perspectives of those who decided against pursuing surgery or a career in academic surgery due to introverted tendencies. Finally, our study deliberately excluded self-identified extroverted participants, as our aim was to explore the experience of introverts. As a result, we cannot draw inferences about extroverted perspectives of academic surgery, including the challenges extroverts face, how those challenges compare to those identified in this study, or how extroverts perceive their introverted colleagues. These questions may be answered through a follow-up study exploring the perspectives of extroverts in academic surgery.

Future mixed method studies should also aim to quantify the rates of introversion and extroversion among academic surgical leaders institutionally and nationally and to qualitatively explore the influence of introversion and extroversion on leadership and management styles. Additional follow-up studies stemming from this study include further evaluating the impact of introversion and related identity dissonance on resident and faculty wellbeing, as well as the impact on resident case volumes, evaluations, and quality of feedback.

## CONCLUSIONS

Although introverted surgeons may experience challenges conforming to cultural norms for professional advancement in academic surgery, their introspective habits, paired with support from role models and mentors, facilitate growth and ultimately, career satisfaction and accomplishment. This study highlights opportunities to better support the development of such introverts in hopes of fostering greater inclusivity within academic surgery.

## AUTHOR CONTRIBUTION

We have more than 8 listed authors on this manuscript. The author contributions are listed as follows: R.T. – conception and design of study, data collection, analysis, and interpretation, writing and revision; S.E.R., P.S. – data analysis and interpretation, revision; J.B.G., C.L.G., G.M.B., R.P., D.C. – data interpretation, revision; S.K.M. – oversight, study design, data interpretation, revision. All authors have reviewed the manuscript and given final approval.

## Supplementary Material
