# Supplementary material for: The Quiet Surgeon: A Qualitative Analysis of the Introverted Experience Throughout a Career in Academic Surgery
Source: Ann Surg Open. 2026 May 29;7(2):e685. doi: 10.1097/AS9.0000000000000685 (PMC13290188; doi:10.1097/AS9.0000000000000685)
Supplement: Supplementary file 1 [file as9-7-e685-s001.pdf]

Supplemental Figure 1. Purposive sampling matrix

**Resident**		
	Man	Woman
Junior (PGY 1-2)	2	2
Senior (PGY ≥3)	2	3
**Attending**		
	Man	Woman
Junior (≤15 YIP)	1	2
Senior (>15 YIP)	3	2

* PGY = post-graduate year, YIP = years in practice
