# Supplementary material for: The Quiet Surgeon: A Qualitative Analysis of the Introverted Experience Throughout a Career in Academic Surgery
Source: Ann Surg Open. 2026 May 29;7(2):e685. doi: 10.1097/AS9.0000000000000685 (PMC13290188; doi:10.1097/AS9.0000000000000685)
Supplement: Supplementary file 2 [file as9-7-e685-s002.pdf]

Supplemental Figure 2. Representative questions from semi-structured interview guide

First, I'd like to explore your relationship with being an introvert.

1. How do you define introversion?
2. How do you know you are an introvert?
3. Overall, do you feel like being an introvert is a positive, neutral, or negative trait?

Next, I'd like to explore the impact of being an introvert on your decision to pursue surgery as a specialty.

4. How did being an introvert impact your decision to pursue surgery as a specialty, if at all?
5. As a medical student, did you have any conceptions of whether surgeons were typically more introverted or extroverted?

Next, I'd like to focus on how introversion impacts your day-to-day life as a resident, and how you may experience some common challenges identified in other spheres of research.

6. What are some of the advantages of being an introvert in surgical training?
7. What are some of the disadvantages of being an introvert in surgical training?
8. As your training has progressed, have you found yourself shifting toward introversion or extroversion?

I'd also like to explore some of your thoughts regarding the impact of introversion on your career trajectory.

9. How, if at all, do you think being an introvert has affected (or will affect) your career trajectory?
10. How, if at all, do you think being an introvert has affected (or will affect) your leadership potential?
11. (If in leadership position) How, if at all, does being introverted impact your leadership style?
12. (If in leadership position) How, if at all, do you adjust your leadership style when interacting with introverted or extroverted team members?

Next, I'd like to ask what advice you would have for…

13. Medical students who are introverts and considering a career in surgery?
14. More junior residents/attendings who are introverted?
15. What advice do you have for attendings or mentors of introverts?

Before we end this interview, I'd like to summarize key ideas we have discussed…

16. (After summary) Is there anything we haven't discussed that you feel is important to know about your experience as an introvert in surgery?
